# Coordination Dynamics: A Foundation for Understanding Social Behavior

**DOI:** 10.3389/fnhum.2020.00317

**Published:** 2020-08-14

**Authors:** Emmanuelle Tognoli, Mengsen Zhang, Armin Fuchs, Christopher Beetle, J. A. Scott Kelso

**Affiliations:** ^1^Human Brain and Behavior Laboratory, Center for Complex Systems and Brain Sciences, Florida Atlantic University, Boca Raton, FL, United States; ^2^Department of Biological Sciences, Florida Atlantic University, Boca Raton, FL, United States; ^3^Department of Psychiatry and Behavioral Sciences, Stanford University, Stanford, CA, United States; ^4^Department of Physics, Florida Atlantic University, Boca Raton, FL, United States; ^5^Intelligent Systems Research Centre, Ulster University, Londonderry, United Kingdom

**Keywords:** HMI, HRI, Coordination Dynamics, social interaction, metastability, multiscale, complex systems, Human Dynamic Clamp

## Abstract

Humans’ interactions with each other or with socially competent machines exhibit lawful coordination patterns at multiple levels of description. According to Coordination Dynamics, such laws specify the flow of coordination states produced by functional synergies of elements (e.g., cells, body parts, brain areas, people…) that are temporarily organized as single, coherent units. These coordinative structures or synergies may be mathematically characterized as informationally coupled self-organizing dynamical systems (Coordination Dynamics). In this paper, we start from a simple foundation, an elemental model system for social interactions, whose behavior has been captured in the Haken-Kelso-Bunz (HKB) model. We follow a tried and tested scientific method that tightly interweaves experimental neurobehavioral studies and mathematical models. We use this method to further develop a body of empirical research that advances the theory toward more generalized forms. In concordance with this interdisciplinary spirit, the present paper is written both as an overview of relevant advances and as an introduction to its mathematical underpinnings. We demonstrate HKB’s evolution in the context of social coordination along several directions, with its applicability growing to increasingly complex scenarios. In particular, we show that accommodating for symmetry breaking in intrinsic dynamics and coupling, multiscale generalization and adaptation are principal evolutions. We conclude that a general framework for social coordination dynamics is on the horizon, in which models support experiments with hypothesis generation and mechanistic insights.

## Introduction

Social systems nest very small structures, the molecular, genetic and cellular machinery of living things, into progressively larger structures – all the way up to entire organisms engaged in mutual interaction with the environment and with each other. Quite crucially and across all levels, the parts (e.g., organelles, organs, organisms, organizations) coordinate dynamically with other parts, engaging and disengaging within and between their respective coalitions and across levels (upward∼downward causation, e.g., genes or neurons influencing social behavior, and vice-versa). A main goal of our research program is to find general systems of equations – expressing lawful regularities – that explain social systems’ coordination dynamics within and across levels, irrespective of level-specific details (Oullier and Kelso, 2009; see also Kelso, 2009a; Kelso et al., 2013). We approach this goal by examining dynamic coordination patterns empirically; embedding those observations in mathematical models; and returning to empirical data to verify newly arisen predictions, in a recursive manner. For present purposes, a model is the recreation of a natural system’s key behavior that facilitates its understanding. “Understanding” is sought, not through some privileged scale of analysis, but within the abstract level of the essential collective variables and their coordination dynamics, regardless of scale or material substrate (Kelso et al., 1987; Schöner and Kelso, 1988b).

Quite a few modeling frameworks have been applied to social systems, including agent-based models (e.g., Axelrod, 1997; Gilbert and Terna, 2000; Bonabeau, 2002; Schweitzer, 2007), cellular automata (e.g., Hegselmann, 1998; Batty, 2007), Lotka-Volterra (e.g., Matsuda et al., 1992; Castellano et al., 2009), stochastic diffusion (e.g., Arató, 2003; López-Pintado, 2008; Kimura et al., 2010), Bayesian (e.g., Yang et al., 2011), Markov (e.g., Singer and Spilerman, 1976; Gintis, 2013), signal flow graphs and block diagrams (Liu and Ma, 2018), recurrent networks (Irsoy and Cardie, 2014), and, central to this review, the HKB model (after Haken et al., 1985; see also Schöner and Kelso, 1988b; Kelso, 1995; Tognoli et al., 2018a), itself based on the concepts of synergetics (Haken, 1977) and the mathematical tools of non-linearly coupled non-linear oscillators. One of the key strengths of the HKB model (and its numerous extensions) is its possession of *intrinsic dynamics* (Kelso, 1995, Ch 6). That is, the system of equations is formalized from two sides: one supplying the intrinsic dynamics of the unit (what it does when left alone to itself), and the other – whose significance social scientists will recognize – reflecting constraints imposed by relation(s) with other units. Intrinsic dispositions and social influences are complementary aspects of social interaction, without which an agent would be carried along by the ebbs and flows of whatever jolts it encounters (see also Friston, 2011 and Kostrubiec et al., 2012 for related views).

Mathematical models, when combined with theoretical concepts, have the power to accomplish an important aim for research that aspires to characterize cross-scale relations: they permit widely different phenomena to fall under common scrutiny. Numerous examples in the history of science speak to the colossal payoff that follows successful integration across scales. Newton’s famous unification of the laws that govern the fall of the apple and the motion of celestial bodies comes to mind. Deterministic chaos and quantum mechanics contain many more examples, although it must be said that “emergent phenomena” exist as well, i.e., where the whole is not only greater than the sum of its parts, but different too (Anderson, 1972; Haken, 1977; Laughlin and Pines, 2000).

How then is one to approach the daunting diversity of dynamical behaviors that is encountered across scales of observation? Our paradigm involves collective variables and non-linear oscillators on the mathematical side, rhythmic finger movements as basic observational units at the behavioral level (Kelso, 1981, 1984; Haken et al., 1985), and of course, neural oscillations at the neural level (see Schöner and Kelso, 1988b; Glass, 2001; Tognoli and Kelso, 2014b). Oscillations might be considered as the “ground zero” of open dynamical systems, their most elemental form. First, temporal symmetries of limit cycles (the mathematical structure for oscillations) allow for fruitful mathematical simplifications. Second, oscillations are pervasive in nature and obvious in their simplest form at the inception of complex organisms: spontaneous oscillations are found in neural and motor activity prenatally (Robertson, 1993; Khazipov and Luhmann, 2006) and destined to endure, albeit in more complex form, throughout the life of living systems (Turrigiano et al., 1994; Gal et al., 2010; Marom, 2010). Evidence for the primordial role of oscillations in human behavior also comes with the occurrence (possibly unmasking) of repetitive movements in several developmental and aging disorders (Brown, 2003; Abbott et al., 2017). There are clear signs that oscillations are exploited for subcortical control (Taga et al., 1991; Grillner et al., 1998; Stewart, 1999; Righetti et al., 2005) and similar hypotheses have been proposed for the cortical level (Edelman and Mountcastle, 1978; Kelso and Tuller, 1984; Yuste et al., 2005; Buzsáki, 2010; Tognoli and Kelso, 2014b). The primacy of oscillations for the regulation and control of living systems has been articulated in the early works of Iberall, Yates, Morowitz, and others. For example, Homeokinetic theory (e.g., Soodak and Iberall, 1978; Yates, 1982) addresses the conditions for persistence, autonomy and self-organization in biological systems from a physical perspective (irreversible thermodynamics). A fundamental tenet is that energy flow from a source to a sink will lead to at least one cycle in the system (Morowitz, 1968). In the homeokinetic view, control is effected by means of coupled ensembles of limit cycle oscillatory processes. Limit cycle oscillations represent the only temporal stability for non-conservative, non-linear systems, that is, they are capable of making up for naturally occurring dissipative losses. Loose coupling of limit-cycle processes exists at all scales. Among their attractive features are their self-sustaining properties, their ability to operate independently of initial conditions, their stability in the face of moderate perturbations, and, perhaps most important the properties of mutual entrainment and synchronization (Minorsky, 1962; Kelso et al., 1981; Winfree, 2001). Furthermore, elaboration of arbitrarily more complex dynamics can be obtained from oscillatory functions, as suggested by the work of pioneer mathematicians like Joseph Fourier, showing some bridges between oscillations and ordinarily irregular dynamics. On both physical and mathematical grounds, therefore, it follows that a path from simple oscillatory dynamics to more complex dynamical behavior (typically observed across multiple scales in biological systems) may be possible.

In the following, we will present briefly our protracted efforts to build more complex dynamics from the paradigm initiated by Kelso and colleagues (see also Avitabile et al., 2016). Before that, we describe briefly the experimental paradigm, analysis strategy, theory and models that constitute the mathematical building blocks of our approach to understanding social coordination. The idea is to show how we grow our modeling paradigm toward increasingly more life-like situations and to identify where the edges of future advances may lie.

## Experimental Paradigm

**FIGURE 1:**
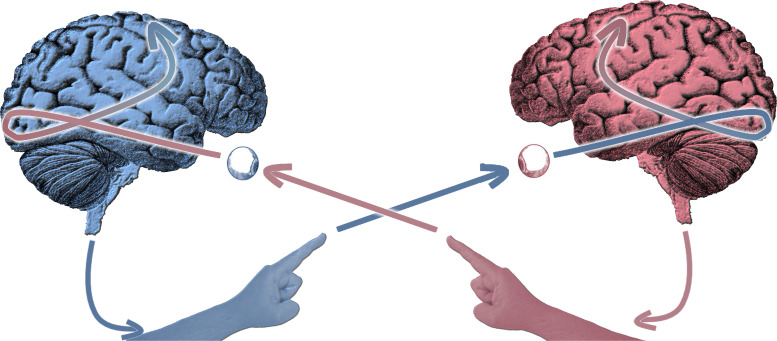
Schematic of the experimental paradigm of social coordination dynamics in which two participants simultaneously perceive and produce behavior in view of each other. More specifically, in our canonical experiment, subjects move their fingers in continuous fashion while at the same time observing their partner doing the same. The paradigm’s simultaneity of dyadic perception and action – bidirectional coupling – is geared toward observing self-organizing processes.

A minimal experimental paradigm for social coordination dynamics has two people exchanging information by virtue of their senses and effectors as shown in Figure 1, forming a figure eight that has maximal symmetry. In our canonical experiments (Tognoli et al., 2007; Oullier et al., 2008; Tognoli, 2008; Tognoli and Kelso, 2015), action is generated by the index finger (often depicted by a phase angle of the finger relative to the joint) and perception is mainly through vision (though both details are certainly amenable to other arrangements within the sensorimotor repertoire of our human subjects, see e.g., Schmidt et al., 1990). This choice does not ignore the fact that plenty of social behaviors (conversations, emotions, dance, jamming, etc.), at first seem to carry much greater sociocultural significance. In the pure tradition of physics, stripping away complications is a strategy to draw out simple controllable pieces of the system and set them in motion in ways that are tractable for mathematical models and their experimental counterparts (see also Kelso, 1995; Stewart, 1999; Nowak, 2004). Our work seeks laws of coordination (subject to proper empirical verifications whenever feasible) that transcend the specific choice of effectors (sweat gland, vocal tract, facial, limb muscles etc.) or sensory pathways in order to draw general mathematical foundations. We start with the simplest dynamics that can be experimentally manipulated and understood theoretically.

With the simple paradigm of two people moving their fingers back and forth in view of one another, we are able to obtain continuous state variables describing the trajectory of each participant’s effector at the behavioral level, their coordination dynamics (viz. the relative phase between the two finger movements, see “Order parameter” below; Tognoli et al., 2007; Oullier et al., 2008; Tognoli, 2008; see also Schmidt et al., 1990, 2011; Richardson et al., 2007; Schmidt and Richardson, 2008; Marsh et al., 2009; Janata et al., 2012; Reddish et al., 2013; Fine and Amazeen, 2014; Fusaroli et al., 2014; Keller et al., 2014; Tschacher et al., 2014; de Poel, 2016; Moreau et al., 2016; for a variety of related approaches). Further, when expanding this work, information can be gained about concomitant activities in the brain (Tognoli et al., 2007; Jantzen et al., 2008; Naeem et al., 2012; see also Hari and Kujala, 2009; Dumas et al., 2011; Sänger et al., 2011; Konvalinka and Roepstorff, 2012; Pfeiffer et al., 2013; Babiloni and Astolfi, 2014; Cacioppo et al., 2014; Hirata et al., 2014; D’Ausilio et al., 2015; Koike et al., 2015; Zhou et al., 2016; Kawasaki et al., 2018; Mu et al., 2018; Pezzulo et al., 2019), and in emotional subsystems (e.g., Zhang et al., 2016; see also Anders et al., 2011; Balconi and Vanutelli, 2017; Reindl et al., 2018). Objective measures of brain and behavior offer tight systems of constraint that connect experiments and experimentally validated models; their continuous nature serves well a modeling framework that uses collective variables/order parameters at the coordinative level and coupled oscillators as components. Finally, such collections of neurobehavioral oscillations aptly embed the multiscale and reciprocal nature of self-organizing processes that play out in social systems (Coey et al., 2012).

## Order Parameter: Connecting Models and Experiments

**FIGURE 2:**
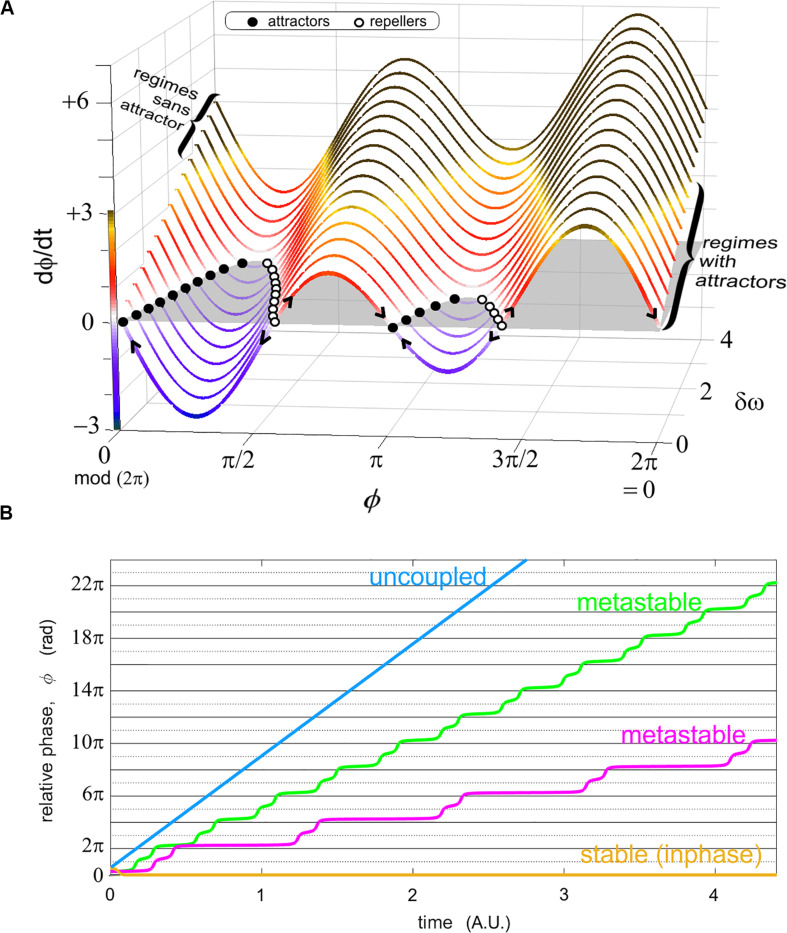
Two representations of the relative phase as an order parameter connecting models and experiments. **(A)** shows the phase portrait of ϕ in the “extended” HKB model (Kelso et al., 1990) for various values of a diversity parameter δω. This graph carries regimes of coordination with attractors in the front of the figure (for modest diversity δω, shown; also when coupling is strong, not shown) and those without attractors (large diversity of the parts, shown in the back of the figure; and/or weak coupling, not shown). Attractors exist when the phase portrait (a function describing the rate of change of the relative phase as a function of itself) has values at $\frac{\text{d}\,\phi }{\text{d}\,t}=0$ (i.e., the coordination does not change over time) and a converging flow (filled black dots attracting the flow as indicated by the arrows). Stable regimes reflect a sustained cooperation among the system’s parts, but this stability also leads to inflexibility (see Kelso and Tognoli, 2007 for more details). In **(B)**, four sample (unwrapped) relative phase evolutions over time illustrate stable coordination (yellow) where the order parameter ϕ persists at the same value (ad infinitum in models); metastability (magenta, green) with their characteristic dwells (quasi horizontal epochs, attracting tendencies near inphase, i.e., 0 rad. and antiphase, i.e., π rad. modulo 2π) and escape (wrapping); and uncoupled behavior (blue), whose relative phase grows continually with time (it approaches a linear function when the probability distribution of individual phases lacks remarkable joint phase ratios).

A key concept of Coordination Dynamics – following along the lines of Synergetics (Haken, 1977) – is the collective variable or order parameter which has been demonstrated to cut across different kinds of parts and processes (and across levels) thereby dissolving traditional (and somewhat arbitrary) divisions (e.g., between “cognitive” and “motor”) and enabling a novel, dynamic framework for understanding collective/social behavior (Kelso, 1995, 2009a,b, 2012; Tognoli, 2008; Coey et al., 2012; Richardson et al., 2014; Tognoli et al., 2018a; see also Macrae and Miles, 2012). In Coordination Dynamics, the order parameter tracks the relation between parts of a system in time (when they come to work together and when they go apart) – that is, the dynamics of their *coordination* in contrast to the dynamics of state variables characterizing the individual component parts. We therefore resort to the relative phase (the difference ϕ between the phase of each oscillator ϕ_*1*_ and ϕ_*2*_), as the collective variable specified in the equations (Figure 2A) and empirically scrutinized in experimental and modeling data (Fuchs and Kelso, 1994, 2009, 2018; Kelso, 1995, 2009a; Lagarde et al., 2006; Kelso and Tognoli, 2007; Tognoli and Kelso, 2009; Fuchs, 2014). From the standpoint of component rhythmic behavior, the HKB model was built from phase-coupled oscillators and accordingly, the theory indicates synchrony or synchronous tendencies as target phenomena (Haken, 2013; see also Strogatz and Stewart, 1993; Bennett et al., 2002; Pikovsky et al., 2002).

Starting in the limit case of strongly coupled systems such as two entrained gears that are so perfectly fit to each other that there isn’t room for either phase trajectory to deviate from the other, the resulting relative phase is constant (Figure 2B, yellow). It is obvious that such a strong and immutable coupling cannot harbor the adaptation that is the hallmark of intelligent living systems, including instabilities, phase transitions and metastability (Kelso, 1995, 2001, 2010, 2012). Complex systems having the necessary propensity for adaptation and reorganization especially entail the latter feature (Figure 2B, magenta, green), that is, parts and coalitions of parts alternate between cooperative tendencies (parts transiently binding, manifested by transiently horizontal epochs, or dwells, of the relative phase) and release from them (phase wrapping; Kelso, 1991). In the other limit when coupling vanishes to zero, the resulting uncoupled system typically manifests a flat diagonal trajectory of its relative phase, reflecting the mere incidence of their respective intrinsic phase dynamics (Figure 2B, blue). Stable trajectories (Figure 2B, yellow) are found in regimes with attractors (Figure 2A, for small values of δω, see right bracket); metastable trajectories in regimes sans attractors (Figure 2A, for larger values of δω past a critical threshold, see left bracket; and weak coupling, not shown).

## Classical Experimental Findings on Social Coordination

**FIGURE 3:**
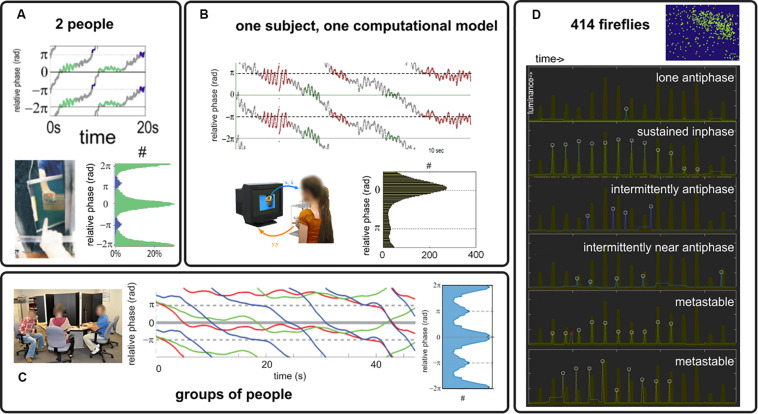
Behavioral coordination in human dyads **(A)**, human interaction with a Human Dynamic Clamp **(B)**, ensemble of people **(C)** and to hint at generality, in fireflies flashing [white circles, **(D)** the thick brown trace indicates the collective behavior of their large colony]. Relative phase trajectories from **(A–C)** are chosen to illustrate an alternation of dwells and escapes that is typical of metastability. Attracting tendencies are found toward inphase and antiphase [see histograms in **(A–C)**, with count identified with the symbol #, showing relative phase distribution for the most strongly coupled conditions in their respective paradigms, see original references for details and for examples of weaker organization].

Under the simple paradigm presented in section “Experimental Paradigm” and with the analysis tools of section “Order Parameter: Connecting Models and Experiments,” we review a number of experimental results supporting the claim that coordination dynamics at multiple scales is metastable, i.e., intermingled dwells and escape in the relative phase exist (relative phase trajectories in Figure 3). We also found what appears to be persistently stable relative phases (not shown, see Tognoli et al., 2007; Kelso et al., 2009; Dumas et al., 2014 for examples), which either pertain to genuine phase locking (dynamical regime *with attractors*) or metastable dwells (*sans-attractor*) whose characteristic time scale exceeds the window size. Findings of metastability span dyadic social coordination (Tognoli et al., 2007; Oullier et al., 2008; Tognoli, 2008; Figure 3A); experiments with a “Human Dynamic Clamp” in which one member of the dyad is a computational model of a surrogate social partner governed by the HKB equations (Figure 3B, Kelso et al., 2009); multiagent social coordination (Figure 3C; Zhang et al., 2018, 2020); and, outside our paradigm but hinting at the phenomenon’s generality, the collective flashing of fireflies (Figure 3D; Tognoli et al., 2018b) that we found not to be fully synchronized *stricto sensu* (recalling that synchronization requires attractors). As predicted from theory, when parts have extensive symmetries in their intrinsic dynamics (small δω, Figure 2A), the attracting tendencies (Figures 3A–C: histograms of the relative phase) have concentrations at inphase (both oscillator trajectories rise and then fall in step) and antiphase (one oscillator rises when the other falls), befitting the extended HKB model as a general model of behavioral and neural coordination (Kelso, 1991; Kelso and Haken, 1995). These observations are also consistent with HKB model predictions (Kelso et al., 1987; Kelso, 2008). For instance, coordination can be weakened by faster dynamics (Kelso et al., 2009), weaker coupling imposed by one participant (as in the “parametrizable” human dynamic clamp: Kelso et al., 2009), or greater diversity of prior dispositions (Zhang et al., 2018, 2020).

In a parallel body of work, we examined the coordination dynamics of brain activity upon which social behavior is predicated, in particular in the domain of electrophysiology. Here again and at several scales, we found evidence of synchronization tendencies or metastability (Tognoli and Kelso, 2009, 2014b). The hypothesis that synchrony (as distinct from synchronization *tendencies*, see below) underlies the binding of local oscillatory processes has taken root in neuroscience (e.g., Gray, 1994; von der Malsburg, 1995; Singer, 1999; Bressler and Kelso, 2001; Varela et al., 2001; Bressler and Tognoli, 2006; Uhlhaas et al., 2009; Buzsáki, 2010; van Wijk et al., 2012; Harris and Gordon, 2015), with the caveat that due to the inherently dipolar nature of electromagnetic fields, electrophysiological data at all scales are replete with spurious inphase and antiphase coordination (Freeman, 1980; Nunez et al., 1997; Nolte et al., 2004; Pascual-Marqui, 2007; Tognoli and Kelso, 2009; Van de Steen et al., 2019). Therefore, empirical evidence needs to be heeded carefully before rendering definitive interpretations of remote synchrony between neural ensembles. Initially setting aside inphase and antiphase (due to the fact that they contain both true and spurious synchronization), we were able to discover transient synchronization patterns (see, e.g., Figure 4 from Tognoli and Kelso, 2009). In the context of our empirical quest to discriminate stability from metastability, synchrony was suggestive of stable states and state transitions–thereby seeming to point to attractors and bifurcations as governing brain dynamics. We however found that a discontinuous spatiotemporal organization was not definitive proof of attractor-based states and transitions in brain dynamics. It is also entirely compatible with the alignment and misalignment of phases across scales that is the hallmark of metastability (e.g., see **Figure 5** from Tognoli and Kelso, 2014b). Moreover, relative phase trajectories, with faint dwells and limited periods of common frequencies, point toward weak coupling. The latter is consistent with the idea that human brains are subject to the coordinative demands of many local populations that trade among each other due to ubiquitous weak coupling. Definitive evidence in favor of either dynamics with or *sans* attractors remains tentative, and its resolution likely resides in perturbation approaches to brain dynamics. Furthermore, because of the pervasive ambiguity of spurious inphase and antiphase in the EEG and the evasiveness of non-inphase dwells from lower scales (Tognoli and Kelso, 2009), it remains difficult to offer definitive evidence of bistable tendencies in brain electrophysiological patterns, and test this specific prediction from the HKB model. Phase transitions, however, have been established and have suggested bistable tendencies in MEG (Kelso et al., 1991, 1992; Fuchs et al., 1992, 2000b) and fMRI dynamics, starting with Meyer-Lindenberg et al. (2002) (see also Fox et al., 2005; Tognoli and Kelso, 2014b).

**FIGURE 4:**
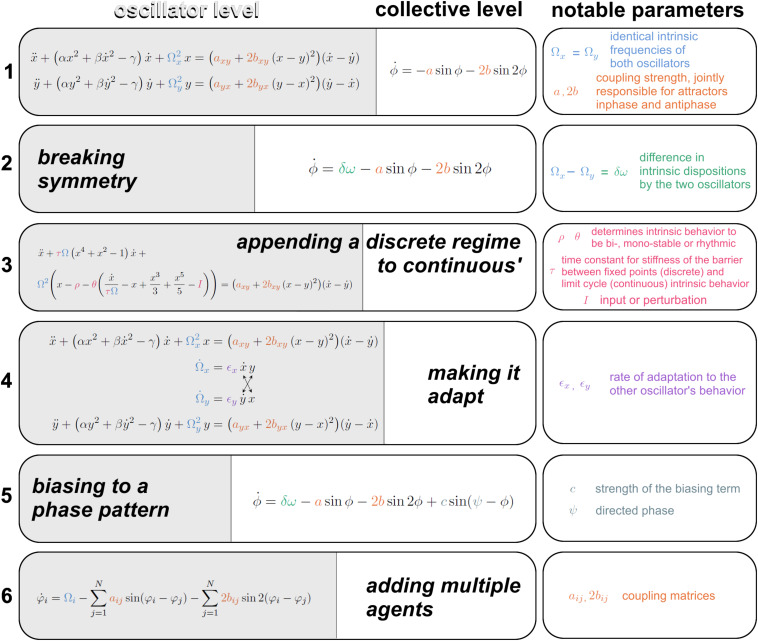
Augmentations of the HKB model toward increasing life-like relevance. **(1)** The first equation is spelled out at the oscillator level on the left (specifying the position, velocity and acceleration of oscillators x and y, with notable parameter Ω carrying the intrinsic frequency, see “Introduction” for motivation); and at a collective level on the right (specifying the rate of change of x and y’s relative phase ϕ. The coupling strength parameters a and b are responsible for the bistability of inphase and antiphase. **(2)** A symmetry breaking term δω (Kelso et al., 1990) gives rise to metastability. **(3)** A modification to the oscillators’ dynamics supplies regimes with discrete behavior -excursion from rest- and continuous cyclical behavior in a single formalism (Jirsa and Kelso, 2005), whose topology is controlled by three parameters ρ, θ, and τ. Transitions between regimes are autonomously followed in a human-machine interaction (Dumas et al., 2014). **(4)** The intrinsic dynamics of the oscillators Ω is coupled with adaptation rate ϵ for partners to adapt their more stable behavioral dispositions as they interact (Nordham et al., 2018). **(5)** A bias parameter Ψ is introduced to direct the coordination to pull human-machine dyads away from their spontaneous attractors with strength c (Dumas et al., 2014). **(6)** A scalable multiagent system of equations is built upon empirical data (Zhang et al., 2019) that carries the first and second order coupling term just like the original HKB model from eq. 1, under coupling matrices a and b. See references in text.

## A Chronology of the HKB Model’s Augmentations

The HKB system of equations was initially developed to model a non-linear, self-organizing phenomenon discovered in human movement coordination (Kelso, 1981, 1984). When simultaneously set in motion, two homologous body parts, for instance left and right hand, may be stabilized in either of two patterns, inphase or antiphase (suggestive of attractors). As movement frequency was increased, however, there was an abrupt switch wherein one pattern lost stability and gave way to the other (here antiphase to inphase). In dynamical system parlance, this switching behavior can be described as an order-to-order phase transition, corresponding mathematically to a bifurcation. The HKB model was built to capture bistability, bifurcation and hysteresis (the finding of different critical frequencies when approaching the bifurcation with decreasing or increasing movement frequencies). Those three elements are characteristic of many complex non-linear systems. Within a multiscale paradigm, it is natural to ask whether governing laws retain their validity at proximal scales (Gell-Mann, 1988; Jirsa and Haken, 1997; Kelso et al., 1999). Our group started to ask whether the HKB equations governing the coordination between two hands of the same person applied when we scale up one level: two hands, one each from two different individuals. Thus, we forayed into social coordination dynamics (Tognoli et al., 2007, 2018a,b; Oullier et al., 2008; Tognoli, 2008; Kelso et al., 2009, 2013; Kelso, 2012; Dumas et al., 2014, 2018; Kostrubiec et al., 2015; Tognoli and Kelso, 2015; Nordham et al., 2018; Zhang et al., 2018, 2019). Social coordination dynamics complemented the same question previously asked at the level of the individual: the hand-owners’ neuro-muscular system studied with neurophysiological tools, MEG, fMRI, and EEG (e.g., Tuller and Kelso, 1989; Kelso et al., 1992, 1998; Wallenstein et al., 1995; Jirsa et al., 1998; Mayville et al., 1999, 2002; Fuchs et al., 2000a,b; Jantzen et al., 2004, 2009; Oullier et al., 2004; Jantzen and Kelso, 2007; Kelso and Tognoli, 2007; Tognoli and Kelso, 2009, 2014b, 2015; De Luca et al., 2010; Banerjee et al., 2012; and many others). Since a sizable body of experimental work had supported HKB’s relevance both in theoretically predicted and *de novo* phenomena (section “Classical Experimental Findings on Social Coordination”), we have adopted HKB as the root model in our social neuroscience research program.

Above, we justified the use of simple behavioral paradigms that were the key to HKB’s initial development. In the following, we demonstrate several advances toward further generalization in the context of social coordination dynamics. Though by no means inclusive^1^, Figure 4 presents six stages of the HKB framework. The original equations (Figure 4, eq. 1; Haken et al., 1985) describe (at the oscillator level on the left and collective level on the right) two non-linearly coupled non-linear oscillators x and y with intrinsic frequency Ω (eigenfrequency), that are bound by a coupling function with notable coupling strength constants a and b. The existence of both coupling terms, one with period ϕ and the second with period 2ϕ, are responsible for the bistability of both inphase and antiphase, and the b/a ratio controls the “depth” of each attractor basin. In the case of eq. 1, Ω_x_ = Ω_y_: the two oscillators have identical intrinsic dynamics, a sensible approximation given the model’s origin with two homologous body parts.

In nature and especially in complex biological and social systems, it is seldom the case that coordination exclusively concerns perfectly matching pieces of machinery: many components ought to work across the divide of different intrinsic dispositions (a child and a father pacing together on a beach; brain areas generating beta and mu oscillations…). The second equation from Figure 4, eq. 2; Kelso et al. (1990) breaks symmetry in the intrinsic behavior of the oscillators, letting Ω_x_ differ from Ω_y_. At the collective level (Kelso et al., 1990; for equations at the component level see Fuchs et al., 1996), this extended HKB model results in a new term δω whose existence has two main effects: it shifts the attractors away from inphase and antiphase coordination and it shrinks the basin of attraction until the weaker antiphase, followed by the stronger inphase, eventually vanish, thereby unlocking a metastable regime that retains attracting tendencies (effective pooling of collective effort), but has lost its fixed points *stricto sensu* (see also Figure 2A). Such metastable regimes are important for their enhanced agility to continually disband coordination patterns and create new ones (Kelso, 1991, 1995; Kelso and Tognoli, 2007).

Obviously from common experience, at some level, functionally adapted systems do not continuously spin the wheel of their dynamics ceaselessly. Numerous processes retain cyclical characteristics under entrainment with the environment or even intrinsically (Glass and MacKey, 1988; Strogatz and Stewart, 1993; Winfree, 2001; Tognoli and Kelso, 2014b; and references in Introduction). But rest is also a characteristic biological behavior; and goal-directed agents, for instance people or brain networks (Attwell and Laughlin, 2001; Kramer and McLaughlin, 2001), tend to set in motion only briefly under intent, perhaps to manage energy constraints (Attwell and Laughlin, 2001). This propensity to switch from cyclical to resting behavior was built into the Excitator model (Figure 4, eq. 3, from Dumas et al., 2014, modified after Jirsa and Kelso, 2005), inspired from the explicitly discrete nature of neuronal excitability (and human movement coordination, Kelso et al., 1979). Its right-hand side (the coupling term) is identical to eqs. 1–2 but its left-hand side has terms that, under parametric control of a separatrix, part the flows of the coordination dynamics into discrete and continuous domains. HKB’s augmentation to discrete dynamics is a building block for many diachronic social behaviors such as turn-taking in talks or mere conspecific observation (e.g., Dumas et al., 2010, 2014; Kawasaki et al., 2013; Tognoli and Kelso, 2015; Pérez et al., 2017) and is intimately linked to delayed coordination and the emergence of roles amongst social participants (Dumas et al., 2014).

Thus far, individual characteristic behavior in isolation has been left unchanged by interaction with others. Now, it is well-recognized in sociology and neuroscience that parts (e.g., people, brain areas) do in fact change (see also Newell et al., 2008), and specifically according to their history of interaction with other parts. This is a truism of adaptation. Taking inspiration from Righetti et al. (2006)’s work on frequency learning in oscillators, Dumas et al. (2014) created an adaptive “Human Dynamic Clamp” based on earlier research on Virtual Partner Interaction (Kelso et al., 2009). In this work, Dumas et al. (2014) used a human to broaden the HKB model’s behavioral repertoire: by making its intrinsic frequency adapt to a human partner’s input, a virtual partner was conditioned to track a slowly evolving range of movements that a human would produce in its “view.” This work opened the way to social adaptation in our mathematical models. It was the first incursion into broader behavioral repertoires for HKB-based surrogate social partners, who grew new capabilities by virtue of their interaction with humans.

In the Dumas et al. (2014) work, however, a relaxation term returns the oscillator to its characteristic frequency preference. Adaptation has one more sweeping phenomenology to hand over to the model and it concerns what happens after, not during the interaction. In the paradigm of dyadic social coordination, Oullier et al. (2008) discerned an increased spectral overlap after episodes of social coordination (“social memory”). Additionally, Tognoli (2008) found the persistence of stable relative phase past the period when visual information exchange secures the binding of both oscillatory trajectories. This aftereffect was further studied by Nordham et al. (2018), who teased apart three factors modulating the strength of social memory: coordinative stability, coupling strength (stronger aftereffects for more stable and more strongly coupled trials) and initial frequency differences (stronger aftereffects in trials with smaller initial differences). It turned out that all three factors arise from a common source. In the modeling section of the same paper, Nordham et al. (2018) showed that a universe of experimental observations in interactional behavior (e.g., strong aftereffect in both partners, in one or in none; multiple precursor conditions before and during interaction that influenced behavior post-interaction) could be accommodated by a single modification of the model. Each oscillator’s intrinsic frequency ceased to be a constant. Instead, it became an equation that conjugated self’s and partner’s dynamics, weighted by an idiosyncratic parameter ϵ – the individual propensity to let one’s self be attracted (or sometimes repelled) by the other (Figure 4, eq. 4). In this adaptive HKB model, social memory deploys all of its forms out of a combination of three parameters: frequency adaptation, coupling strength and initial frequency difference.

Now let’s consider the phase pattern at which social coordination happens. While experimental data demonstrate that effortless coordination between two people occurs at or near inphase and antiphase (Section “Classical Experimental Findings on Social Coordination” and Schmidt et al., 1990; Richardson et al., 2007; Marsh et al., 2009; Fine et al., 2015; though see Avitabile et al., 2016 for nuanced theoretical and empirical insights), there is plenty of skillful interpersonal coordination that ought to happen against the grain of “natural” tendencies, e.g., in orchestra playing (Walton et al., 2015) or in the performance of skillful joint actions (Duarte et al., 2012; Issartel et al., 2017). An experimental line of research on learning had shown the remodeling of attractive states/tendencies as they are subjected to practice (Schöner and Kelso, 1988b; Zanone and Kelso, 1992, 1997; Kelso and Zanone, 2002; Kostrubiec et al., 2012, 2015). Change in the attractor landscape was modeled by Schöner and Kelso (1988a) as a task requirement (“informational forcing”) that tuned the locus of the attractors. Considering that conspecifics are a crucial part of the environment (MacMahon et al., 1978; Dunbar and Shultz, 2007; Barsalou, 2008; Adolphs, 2009), Dumas et al. (2014) expanded the additional forcing term from Schöner and Kelso (1988a) into a Virtual Partner parameter to lure humans into collective behaviors that would *a priori* be unstable for human dyads to perform. To do so, the Virtual Partner’s mathematical model was augmented with a biasing term that attracted the collective dynamics to a target phase Ψ with strength C (Figure 4, eq. 5). Incorporating this feature into the Human Dynamic Clamp, Kostrubiec et al. (2015) demonstrated that spontaneously unstable patterns of phase coordination (e.g., at 90°) could be coproduced by a human and a Virtual Partner, the latter set with a strong bias to teach that pattern to the human. Some degree of learning was also corroborated, and the system was nicknamed “Virtual Teacher.” This model augmentation ventures into social contexts where the participants’ roles are markedly different: a computationally forged bias (representing the timescale of attracting structures) allows transfer of coordinative patterns between participants, characteristic of social learning.

All the models exposed above pertain to dyadic situations. Early on in Coordination Dynamics, the question of multiagent models had been addressed by Schöner et al. (1990) and Jeka et al. (1993) in the context of “quadrupedal” coordination patterns of people’s upper and lower limbs in which the (collective) state variables of the system constitute 3 relative phases. But proposals to scalable n-dimensional HKB systems remained to be achieved. Zhang et al. (2018) reasoned that useful foundations rested with empirical data at the intermediate scale (somewhat bigger than two to make room for groupings within groupings, but smaller than the experimentally unattainable infinity; and importantly, tractable for a detailed analysis of phase coordination patterns). To constrain the model with empirical data (and with the underlying goal of integrating across levels, see “Introduction”), Zhang et al. (2018) set up the perceptual-motor coupling of eight people whose movements caused taps on a touchpad and who saw everyone’s taps as flickering patterns on spatially situated LED arrays (Figure 3C). The subsequent model development explored a variety of frameworks before settling on a hybrid system of HKB and Kuramoto equations (Zhang et al., 2019; Figure 4, eq. 6; including a coupling term with connectivity matrix a_ij_ fully equivalent to the Kuramoto model and another term with connectivity matrix b_ij_ resembling the second order coupling term from HKB, see eqs. 1,2,5). Development of the model built on useful functional symmetries carefully crafted in the experimental setup. The combination of extended HKB and Kuramoto fulfilled all the empirical constraints provided by Zhang et al. (2018) across multiple levels of description. Importantly, the Kuramoto model alone was insufficient because it did not uphold co-expressed inphase and antiphase patterns that are central features of HKB and that the eight agent experiment had uncovered. With this scalable system now made relevant by experimental data on human multiagent coordination, an opportunity exists to relate coordination dynamics across multiple scales, and especially across levels of description spanning the neural, behavioral and social [for an interesting related approach that also uses HKB coupling, see the ‘sheep herding’ paradigm of Nalepka and colleagues (Nalepka et al., 2017, 2019)].

## Conclusion and Outlook

To complement many approaches that focus on a unique level of description of social behavior and to gain insight into the relationship between scales, we asked whether the multitudinous processes associated with social behavior abide to general principles. In section “Classical Experimental Findings on Social Coordination” of this review, we presented a series of experimental snapshots taken from two levels (brain and behavior), all pointing to spatiotemporal metastability as a common organizing principle. Metastability arises from weak coupling (permissive of flexible binding) and diversity (tendency of the parts to unbind and act independently) – the tension between the two opens up a universe of complex coordinative patterns besides phase-locking. Metastability is especially well understood in its simpler forms near the border of the bifurcation from stable phase-locking (Kelso, 1991, 2012; Zhang et al., 2018). Furthermore, metastability probably remains in effect throughout more complex coordination patterns, even as we may fail to identify and quantify its more elusive forms (Tognoli and Kelso, 2014a; see also Zhang et al., 2020 for methodological advances using computational algebraic topology).

Recognizing that evolutionarily speaking, rhythm is a powerful way to put people in tune with one another (whether it be to dance, to compete, to make war, or to worship), we have taken oscillatory dynamics as the workhorse of our research paradigm. Yet, oscillatory behavior is all but its terminal end game. In section “A Chronology of the HKB Model’s Augmentations,” we laid out the state of our progress to bring the above experimental insights of social coordination into an evolving modeling framework: starting from a pair of symmetrical non-linearly coupled non-linear oscillators (Figure 4, eq. 1), crossing the crucial step of broken symmetry that unleashes metastability (eq. 2), and augmenting for discreteness (eq. 3), frequency adaptation (eq. 4), intentionally directed phase patterning (eq. 5), and finally scaling for multiple interacting agents (eq. 6) as a leap into a multiscale framework. These augmentations progressively evolve a repertoire of coordinative behaviors with increasing realism: elaborating on the intrinsic dynamics, coupling and within- and across-scale composition in a self-consistent manner. They echo numerous calls for models that reach beyond the original HKB model (Jirsa et al., 1998; Beek et al., 2002; Newell et al., 2008; Kelso et al., 2013; Avitabile et al., 2016; de Poel, 2016; Słowiński et al., 2018, 2020) and lay some foundation for generative approaches to the complexity of intrinsic and social dynamics that our interdisciplinary group continues to pursue.

Grounded in the dynamics of sensorimotor loops that couple perception and action between two or more individuals (Figure 1), more profound sociocognitive concepts quickly emerge. As posited in the introduction, an essential characteristic of the present modeling framework is that it approaches social behavioral dynamics from two standpoints: one for the intrinsic dynamics of the self and one for the coupling to the partners, their socialness. The equations governing the evolution of self-behavior (Figure 4, left hand-side of leftmost column) are dynamical mechanisms that intertwine self and others via the interaction (right hand-side of same). However, for the self to remain distinct from, yet informed by social partners, there needs to be a separation of time scales at which self-disposition and input from others influence individual behavior. Our model of social memory (Figure 4, eq. 4), contains distinct time scales and coupling for the moment-to-moment coordination of behavior (Figure 4, eq. 4, parameters a and 2b, purple color), and for the influence that the other(s) exert(s) more permanently on self-dispositions (Figure 4, eq. 4, parameter epsilon, red). If it is a key asset of biological adaptation to modify one’s internal state (Maturana, 1970), then our work highlights how neurobehavioral symmetries at play in social interaction contribute to shaping human behavior (see also Dumas et al., 2012), a well-recognized concept in sociology, developmental psychology and learning science (Thelen and Smith, 1994; Mercer, 2011; Sheets-Johnstone, 2017).

From the intricacies of self and others (above) immediately follows the question of agency (Kelso, 2000, 2016; De Jaegher and Froese, 2009; Buhrmann and Di Paolo, 2017; Solfo et al., 2019). In a line of the model, we demonstrated how to stabilize initially unstable phase patterns using the equations for directed coordination (Figure 4, eq. 5; Dumas et al., 2014; Kostrubiec et al., 2015), which was transformed into an agentic learning tool in human machine interaction. On the subjective side, Kelso (2016) has theorized that a developmental phase transition to agency occurs when infants realize the impact of their action on the world. Kelso and Fuchs (2016) have developed a model of this phase transition. The question of agency reaches an apex of complexity when multiple intentionalities are conflated into a collective outcome. In our study of the Human Dynamic Clamp, we have shown that the model is able to tune various aspects of its coupling strength to modify the rate at which it converges to its “intention” and therefore gain or lose agency to a competitive human partner whose temporal dynamics is probably more constrained than the model’s (Kelso et al., 2009; Dumas et al., 2014). A study of human brain (Dumas et al., 2020) suggests that the subjective sense of agency arising within such human-machine interactions has its root in the neural dynamics entrained by the movements from self and other, whose convergence occurs in the right parietal cortex. This set of results not only highlights the key role of right parietal areas in social coordination, but also points toward a link between sensorimotor neuromarkers and affective dimensions of human social cognition (see also Zhang et al., 2016), in agreement with paleocognitive accounts of the right hemisphere as an evolutionary neuroanatomical basis from predatory threat avoidance to social processing (Forrester and Todd, 2018). It is hoped that the present work may eventually speak to higher level processes such as the mentalizing versus simulation debate in social cognition (Gallagher, 2008; Frith and Frith, 2012; Sperduti et al., 2014; Alcalá-López et al., 2019).

A multiscale framework sufficiently mature to encompass sensory, cognitive and motor abilities will further allow one to explore the effect of traits and pathologies on coordinative competencies. We showed that the model cross-validates with experimental studies when parametric manipulations predictably induce phase transitions (i.e., a logic distinct from curve-fitting). The social coordination dynamics framework may overcome the curse (for the scientist, since functionally, it is a blessing) of behavioral “degeneracy” [equivalence of behavioral coordinative (dis)abilities arising under distinct individual sensorimotor organizations] by dissecting neurobehavioral roots of social behavior in conditions such as autism or Parkinson’s Disease (see also Lagarde, 2013; Dodel et al., 2020 for related views). Specific experiments across traits and conditions, guided by modeling insights, also power neurobehavioral diagnostic tools with great specificity (e.g., Baillin et al., 2019).

Our most recent innovation with multiple agents complementing the prior dyadic formalism (Figure 4, eq. 6; Zhang et al., 2019) has provided a decisive stepping-stone for the multiscale framework that has long been envisioned. By marrying models of coordination based on statistical mechanics (the Kuramoto model, Kuramoto, 1984) and non-linear dynamics (extended HKB, Kelso et al., 1990), the Zhang et al. (2019) model from eq. 6 provides an experimentally validated framework where coordinative structures can exist within other coordinative structures – the ground zero for vertical integration across scales. From a complex systems perspective, this is a much-needed innovation because external control elements (the parameters that scientists tune and set) can now be incorporated by layering systems within systems, with the immediate consequence that the loose ends previously left in the hands of scientists can be returned to self-organizing principles and advance increasingly autonomous architectures recapitulating social behavior across scales. A neurocomputational model of social behavior (Dumas et al., 2012; Tognoli et al., 2018a) is but one of them. The development of this scalable, empirically validated framework also allows one to examine multiscale coordinative structures and study how they arise from simple (but no simpler) interaction between individuals. In particular, by introducing more “space” (degrees of freedom), this framework generalizes the impact of metastability, a mechanism originally discovered in dyadic interaction, to a system level: it creates spatiotemporal metastability, allowing a large-scale system to adopt very many different configurations in a sequential, recurrent manner. In other words, metastable coordination dynamics endows a system with an ability to generate complex, yet organized, spatiotemporal patterns – the sign of a true complex system.

## Author Contributions

All authors conceived the review presented in the manuscript. ET, MZ, CB, and JK contributed to the final manuscript.

## Conflict of Interest

The authors declare that the research was conducted in the absence of any commercial or financial relationships that could be construed as a potential conflict of interest.

## Footnotes

1 For example, other developments not considered here include learning, recruitment of new degrees of freedom, parametric stabilization, multifrequency coordination, trajectory formation, stabilization of unstable systems, intentional switching, etc. Most of these aspects have been developed within single subject situations and have yet to be systematically explored in social contexts, our focus here. They do, quite obviously, provide a reservoir for future work in social coordination dynamics.
